# The Relationship Between Climate Change Worry and Symptoms of Stress, Anxiety, and Depression in Turkish Pregnant Women: A Cross-Sectional Study

**DOI:** 10.3390/healthcare14040493

**Published:** 2026-02-14

**Authors:** Selver Bezgin, Sibel Akgül Kartal

**Affiliations:** 1 Department of Mental Health and Diseases Nursing, Faculty of Health Sciences, Van Yüzüncü Yıl University, Tuşba 65040, Van, Türkiye; 2 Department of Midwifery, Faculty of Health Sciences, Van Yüzüncü Yıl University, Tuşba 65040, Van, Türkiye; sibelakgulkartal@yyu.edu.tr

**Keywords:** pregnancy, climate change, worry, anxiety, depression, stress

## Abstract

**Background/Objectives:** This study aimed to examine the relationship between climate change worry and symptoms of stress, anxiety, and depression in pregnant women. **Methods:** The cross-sectional study was conducted with 367 pregnant women. Data were collected using the “Personal Information Form,” the “Climate Change Worry Scale (CCWS),” and the “Depression Anxiety Stress Scale (DASS-21).” Spearman rho and Kruskal–Wallis-H Test were used to analyze the data. **Results:** The mean total score for the CCWS was 20.22 ± 8.20. Significant differences in the scores for the CCWS were found for the levels of education, economic income, stated concern regarding climate change, and perceived effects of climate change for their location (*p* < 0.05). Significant positive correlations also became apparent between the overall scores and subscale scores for the CCWS and the DASS-21 (*p* < 0.001). **Conclusions:** In conclusion, the findings of this study indicate that climate change concern is significantly associated with depression, anxiety, and stress among pregnant women.

## 1. Introduction

Climate change is an international problem that includes natural fluctuations in the climate as well as changes that occur in the atmosphere due to human intervention [1]. This problem poses multiple threats, not only to the environment but also to society, the economy, and public health. The World Health Organization has projected that between 2030 and 2050, climate change will cause around 250,000 additional deaths annually from malnutrition, malaria, diarrhea, and heat-related diseases [2]. Research indicates that climate change affects not only physical health but also mental health [3,4,5,6,7]. Some groups, such as children, the elderly, and pregnant women, are particularly vulnerable to these impacts [7,8].

In recent years, terms like climate grief, solastalgia, and eco-anxiety have emerged in the literature to describe the emotional reactions to environmental catastrophes, such as climate change, and their impact on mental health [4,5,9]. Solastalgia refers to the distress individuals feel when their immediate environment is being destroyed, leading to feelings of disorientation and a sense of not belonging to that place [10,11]. The concept of climate grief refers to the profound sadness people experience due to the environmental losses brought on by climate change [12]. Eco-anxiety is a term that captures concerns about future environmental threats, particularly climate change [4,13]. Although terms such as eco-anxiety, climate change anxiety, climate change worry, and environmental anxiety are often used interchangeably, some studies suggest that eco-anxiety could be viewed as a broader concept, with climate change worry or climate change anxiety as potential subdimensions [14,15].

Pregnancy is a unique period when women experience increased physical and emotional sensitivity. During this time, concern about climate change is particularly relevant, as expectant individuals often consider how environmental threats will affect not only their health but also the future of their child. As such, the emotional impact of climate change worry may be experienced differently and with dual effects during pregnancy [3,16,17,18]. Recent studies have increasingly focused on the mental health implications of climate change-related worry during pregnancy. These studies suggest that climate change concern during pregnancy may contribute to mental health challenges, including anxiety and depression, both before and after childbirth [16,18]. While research on this topic is limited in Turkey, existing studies have yielded significant findings. For example, one study found that concern about climate change was a significant predictor of depressive symptoms among pregnant women [19]. Another study identified a positive association between climate change anxiety and quality of life in pregnant women, noting that anxiety levels tended to increase with higher levels of education [20].

Understanding the psychosocial impacts of climate change on pregnant women is crucial, not only for supporting mental health on an individual level but also for informing effective environmental policies and designing preventive health interventions [8]. The study aimed to examine the relationship between climate change worry and symptoms of stress, anxiety, and depression in pregnant women. To achieve the aims of the study, the research questions were determined as follows:

1. What is the level of climate change-related worry among the participants?
2. Do levels of climate change-related worry significantly differ based on the participants’ defining characteristics?
3. Is there a significant relationship between participants’ worry about climate change and symptoms of stress, anxiety, and depression?

## 2. Materials and Methods

### 2.1. Design of Study

The study employed cross-sectional research approach.

### 2.2. Participants

The study population consisted of approximately 815 pregnant women who applied to the Obstetrics and Gynecology Outpatient Clinic of a training and research hospital over a five-month period. The study sample comprised 367 pregnant women. Post hoc power analysis with G*Power 3.1 indicated that the statistical power (1-β) was 1.00 (100%) at an alpha level of 0.05 and an effect size (d) of 0.64, confirming the adequacy of the sample size.

When the participants’ age and educational levels were examined, it was found that 54.5% were between the ages of 26 and 35; regarding their educational background, 22.3% had completed primary school, 24.3% secondary school, 28.1% high school, and 25.3% had a university degree or higher. In terms of gestational age distribution, 60.2% were in the third trimester. Regarding participants’ concerns about climate change and their expectations about the potential impact on their region, 48.2% reported that they did not experience any concern related to climate change during pregnancy, while 40.6% stated that they believed their region would be somewhat affected by climate change (Table 1).

#### Inclusion and Exclusion Criteria

Inclusion criteria included a minimum age of 18 years and the ability to read and write. Maternal or fetal risk factors such as preeclampsia, cardiac disease, placenta previa, oligohydramnios, and fetal anomaly were determined as exclusion criteria in the study to minimize potential confounding effects on psychological symptoms.

### 2.3. Data Collection

The study was conducted with 367 pregnant women who presented to the obstetrics and gynecology outpatient clinic of a training and research hospital and met the study criteria. After obtaining ethical approval and institutional permission, the study was carried out between February 2025 and July 2025. Participants were recruited using a convenience sampling method. Data were collected by the researchers through face-to-face interviews in a private room within the outpatient clinic. Completion of the Personal Information Form and the relevant measurement tools took approximately 10–15 min per participant. Considering the participants’ voluntary participation, the exact number of women who were informed about the study but declined to participate is unknown, but it is estimated to be between 40 and 60. Furthermore, because the data collection tools used in the study were very brief, there was no missing data.

### 2.4. Measurements

Data were collected using the “Personal Information Form,” the “Climate Change Worry Scale (CCWS),” and the “Depression Anxiety Stress Scale (DASS-21).”

The Personal Information Form

This form, developed by the researchers, includes demographic information such as the participants’ age, educational status, monthly income level, gestational week, and number of pregnancies. In addition, it contains questions regarding whether the participants experienced concern related to climate change during pregnancy and their expectations about whether the region they live in will be affected by climate change.

The Climate Change Worry Scale (CCWS)

The scale was developed by Stewart [21] to assess the frequency of concern related to climate change. It was later adapted for the Turkish population by Gezer and İlhan [22]. The CCWS consists of 10 items, and participants rate each item on a 5-point Likert scale (1 = never, 2 = rarely, 3 = sometimes, 4 = often, 5 = always). The scale comprises two subscales: anxiety (e.g., Even though the effects of climate change seem far off, hearing about them makes me feel anxious) and feeling of helplessness (e.g., I feel worried that I won’t be able to cope with climate change). It should be noted that the ‘anxiety’ subscale of the CCWS assesses worry specifically related to climate change, whereas the DASS-21 anxiety subscale measures broader symptoms of clinical anxiety. In the reliability analysis conducted by Gezer and İlhan [22], the Cronbach’s alpha internal consistency coefficient for the overall scale was found to be 0.91. The Cronbach’s alpha coefficients for the anxiety and feeling of helplessness subscales were 0.87 and 0.83, respectively [21,22]. In the current study, the Cronbach’s alpha coefficient for the overall scale was found to be 0.922, while the coefficients for the anxiety and feeling of helplessness subscales were 0.884 and 0.814, respectively.

The Depression Anxiety Stress Scale (DASS-21)

The 42-item Depression Anxiety Stress Scale (DASS-42), developed by Lovibond and Lovibond [23], is a self-report instrument designed to assess individuals’ symptoms of depression, anxiety, and stress [23]. Following that, several studies created a 21-item scaled-down form, the DASS-21, consisting of seven items per subscale [24,25,26,27,28]. The DASS-21 was also determined to have adequate measurement properties among diverse groups of people [24,25,26,27,28,29,30,31]. The short 21-item version of the scale used in this study was adapted for the Turkish population by Yılmaz et al. [31]. The scale is rated on a 4-point Likert-type scale (0 = does not apply to me at all, 1 = applies to me to some degree, 2 = applies to me to a considerable degree, 3 = applies to me very much). There are three sections, and each contains seven items: depression (e.g., I felt I had nothing to look forward to), anxiety (e.g., I felt afraid for no reason), and stress (e.g., I found it difficult to unwind). The score for each section ranges from 0 to 21, with higher scores indicating more severe psychiatric distress. Cronbach alpha coefficients for the short Turkish form of the scale were also determined to be 0.808 for the subscale for anxiety, 0.819 for the subscale for depression, and 0.755 for the subscale for stress [31]. The Cronbach’s alpha coefficients were found to be 0.798 for anxiety, 0.839 for depression, and 0.866 for stress in this study. The subscales of the Dass–21 were categorized into five levels: normal, mild, moderate, severe, and extremely severe. Accordingly, for the depression subscale, scores were classified as 0–4 normal, 5–6 mild, 7–10 moderate, 11–13 severe, and 14 and above extremely severe. For the anxiety subscale, scores of 0–3 were considered normal, 4–5 mild, 6–7 moderate, 8–9 severe, and 10 and above extremely severe. For the stress subscale, scores of 0–7 were evaluated as normal, 8–9 mild, 10–12 moderate, 13–16 severe, and 17 and above extremely severe [32].

### 2.5. Data Analysis

Statistical analysis for IBM SPSS Statistics for Windows (Ver. 23.0) was carried out with significance set at *p* < 0.05. The first step is to establish whether the distribution of the data is suitable for the effective use of statistical procedures. The Kolmogorov–Smirnov test for normality showed that the data were not normally distributed (*p* < 0.05). Based on this result, Kruskal–Wallis H was used to look at the differences in group means when comparing three or more groups. In pairwise comparisons (post hoc) conducted to determine the source of differences between groups, the Dunn test with Bonferroni correction was applied to prevent multiple comparison error. Epsilon-squared effect size (ϵ^2^) coefficients were calculated to interpret the magnitude of the obtained differences. Spearman rho analysis was performed to examine the relationships between continuous variables.

### 2.6. Ethical Approval

Prior to the commencement of the study, ethical approval was obtained from Van Yüzüncü Yıl University (dated 20 September 2024 and numbered 2024/10-38) as well as institutional permission (dated 6 February 2025 and numbered E-50817530-771-268255186). Before data collection, participants were informed about the purpose and procedures of the study. Verbal and written informed consent was obtained from those who agreed to participate voluntarily, in accordance with the Declaration of Helsinki, revised in 2013 in Brazil.

## 3. Results

Participants’ mean scores on the Climate Change Worry Scale (CCWS) and the Depression Anxiety Stress Scale (DASS-21) were analyzed. Accordingly, the mean total score on the CCWS was 20.22 ± 8.202, with a mean of 14.26 ± 5.686 on the CCWS anxiety subscale and 5.96 ± 2.806 on the CCWS helplessness subscale. The mean scores for the subscales of the DASS-21 were as follows: 3.84 ± 3.850 for depression, 6.10 ± 4.188 for anxiety, and 5.12 ± 4.578 for stress (Table 2).

Table 3 presents the analysis results comparing the mean rank scores of CCWS and its subscales based on the participants’ descriptive characteristics. Statistically significant differences were found in total CCWS scores according to education level (χ^2^_(df=3,n=367)_ = 16.930; *p* = 0.001), perceived economic status (χ^2^_(df=2,n=367)_ = 7.308; *p* = 0.026), level of concern about climate change during pregnancy (χ^2^_(df=3,n=367)_ = 47.052; *p* = 0.000), and expectations regarding the impact of climate change in their region (χ^2^_(df=3,n=367)_ = 53.035; *p* = 0.000). Specifically, participants with a university degree or higher had significantly higher CCWS total scores than those with lower education levels (*p* < 0.05). Similarly, women who perceived their income to be greater than their expenses scored higher than those who reported equal or insufficient income (*p* < 0.05). Participants who reported moderate or high levels of concern about climate change during pregnancy had significantly higher total CCWS scores compared to those who reported little or no concern (*p* < 0.05). Furthermore, women who believed their region would be severely affected by climate change had significantly higher scores than those who expected no or minimal impact, or had no opinion (*p* < 0.001). In the anxiety subscale of CCWS, significant differences were also observed based on education (χ^2^_(df=3,n=367)_ = 16.720; *p* = 0.001), economic status (χ^2^_(df=2,n=367)_ = 7.459; *p* = 0.024), concern about climate change during pregnancy (χ^2^_(df=3,n=367)_ = 45.262; *p* = 0.000), and regional impact expectations (χ^2^_(df=3,n=367)_ = 52.063; *p* = 0.000). Women with a university degree or higher, as well as those with higher perceived income, reported significantly higher climate change-related anxiety scores (*p* < 0.05). Likewise, those with greater concern about climate change or who anticipated significant regional impacts had higher climate change-related anxiety scores than their counterparts (*p* < 0.001). For the feeling of helplessness subscale of the CCWS, significant group differences were found in relation to education (χ^2^_(df=3,n=367)_ = 13.712; *p* = 0.003), concern about climate change during pregnancy (χ^2^_(df=3,n=367)_ = 41.663; *p* = 0.000), and regional impact expectations (χ^2^_(df=3,n=367)_ = 46.220; *p*= 0.000). Similarly to other findings, university-educated women and those with higher concern levels reported greater feeling of helplessness scores (*p* < 0.05 and *p* < 0.001, respectively). Participants who expected their region to be highly affected also had significantly higher feeling of helplessness scores than those who expected no or minimal impact or had no opinion (*p* < 0.001). No statistically significant differences were observed in total CCWS or its subscales based on age, gestational week, or number of pregnancies (*p* > 0.05). Additionally, perceived economic status was not significantly associated with feeling of helplessness subscale scores (*p* > 0.05) (Table 3).

Table 4 summarizes the comparison of mean DASS-21 scores according to the descriptive characteristics of the participants. Accordingly, a statistically significant difference in the depression subscale scores was found across groups only in relation to educational status (χ^2^_(df=3,n=367)_ = 14.132; *p* = 0.003) and participants’ expectations regarding the impact of climate change in their region (χ^2^_(df=3,n=367)_ = 13.911; *p* = 0.003). It was found that the depression subscale scores of women who graduated from high school were higher than those of women who graduated from secondary school (*p* < 0.05). It was found that women who had no opinion regarding whether the region they lived in would be affected by climate change had higher depression subscale scores compared to other women groups (*p* < 0.001). Regarding the anxiety subscale scores, statistically significant differences were found between the groups based on the number of pregnancies (χ^2^_(df=2,n=367)_ = 7.358; *p* = 0.025). Accordingly, women with their first pregnancy had higher scores on the anxiety subscale than women with 2–3 and 4 or more pregnancies (*p* < 0.05). Regarding the stress subscale scores, a statistically significant difference was found in relation to the number of pregnancies (χ^2^_(df=2,n=367)_ = 7.336; *p* = 0.026) and participants’ expectations regarding the impact of climate change in their region (χ^2^_(df=2,n=367)_ = 8.356; *p* = 0.039). Accordingly, women experiencing their first pregnancy had higher stress subscale scores than those in their second or third pregnancy (*p* < 0.05). It was determined that the mean rank of women who have no opinion how their region is affected by climate change had higher stress subscale scores compared to those who thought their region would not be affected or would be affected only somewhat (*p* < 0.005). In contrast, the analyses revealed no statistically significant difference in the depression, anxiety and stress subscales of the DASS-21 based on age, economic income status, gestational week, and concerns about climate change during pregnancy (*p* > 0.05) (Table 4).

Table 5 presents the results of Spearman’s rho correlation analysis conducted to examine the relationship between the CCWS total and its subscales and the DASS-21 subscales. According to the results, significant positive and moderate relationships were found between the CCWS total and the DASS-21 subscales of depression (r = 0.360), anxiety (r = 0.387), and stress (r = 0.327) (all *p* < 0.001). In addition, significant positive and moderate relationships were found between the anxiety subscale of CCWS and the DASS-21 subscales of depression (r = 0.326), anxiety (r = 0.390), and stress (r = 0.318) (all *p* < 0.001). There were also significant positive and moderate correlations between the helplessness feelings dimension of the CCWS and the DASS-21 subscales for depression (r = 0.373), anxiety (r = 0.334), and stress (r = 0.298) (all *p* < 0.001) (Table 5).

## 4. Discussion

This study examined the association between climate change worry and symptoms of stress, anxiety, and depression among pregnant women. To contextualize participants’ psychological status, mean scores on the DASS-21 subscales were evaluated. Based on these scores, depression and stress were generally within the normal range, whereas anxiety may be considered moderate. These findings may indicate that clinically significant depressive or stress symptoms were uncommon, while a moderate level of anxiety could be present in the sample. Overall, participants reported moderate levels of climate change worry, with anxiety-related responses on the CCWS exceeding feelings of helplessness. This pattern is consistent with previous research identifying anxiety, worry, and sadness as the most common psychological responses to climate change [34,35,36].

Regarding participant characteristics, individuals with a university degree or higher, as well as those whose income exceeded their expenditures, exhibited elevated levels of climate change worry. This finding aligns with prior studies indicating that higher educational attainment and income are associated with greater awareness of and emotional engagement with climate-related issues [20,37,38,39,40]. Furthermore, participants who perceived climate change as a threat to their local environment reported higher climate change worry scores, supporting the notion that perceived proximity to environmental risks intensifies emotional responses [41]. While the analyses revealed associations between CCWS scores and climate change worry and expectations that the region will be affected by it, these relationships should be interpreted with caution due to conceptual overlap with the scale itself. This analytical circularity may limit the explanatory value of these findings. However, these analyses were included to provide preliminary insight into the convergent validity of the CCWS in this population.

Nearly half of the participants (48.2%) reported no concern about climate change worry, which could indicate that this subgroup had relatively low awareness of, or perceived personal relevance regarding, the issue. Previous studies conducted in Turkey suggest that public awareness of environmental problems is generally limited [42,43,44]. This pattern may have contributed to the relatively low mean levels of climate change worry observed in the sample and to the apparently weaker associations between climate change worry and psychological symptoms.

Moreover, participants with heightened climate change worry also obtained higher scores on the helplessness and anxiety subscales of the CCWS, consistent with earlier findings [18,45]. In contrast, demographic variables such as age, gestational week, and number of pregnancies were not significantly associated with climate change worry. This suggests that factors beyond basic demographic characteristics—such as direct exposure to environmental risks, frequency of information exposure, individual interpretations of climate change, social support networks, and cultural context—may play a more substantial role in shaping climate change worry during pregnancy [46,47,48,49].

Beyond these findings, it is important to consider why climate change worry may be particularly pronounced during pregnancy. Pregnancy represents a critical life transition characterized by heightened emotional sensitivity, biological changes, and increased future-oriented concerns. By its nature, climate change worry is future-focused, and for pregnant women, this future is often linked to the well-being of the unborn child [16,17]. Moreover, this process may be shaped not only by the biopsychosocial characteristics of pregnancy but also by the cultural context. In societies such as Turkey, where traditional gender roles are predominant, childcare and caregiving responsibilities are largely ascribed to women, potentially increasing pregnant women’s sense of responsibility for their children’s current and future well-being [50,51]. Within this cultural framework, the traditional gender roles ascribed to women may contribute to elevated stress and emotional burden during pregnancy [50]. When combined with concerns about climate change—a global and largely uncontrollable threat—these culturally reinforced responsibilities may further exacerbate psychological distress among pregnant women.

Further analyses revealed moderately significant positive correlations between total CCWS scores (and its subdimensions) and all three DASS-21 subscales: depression, anxiety, and stress. Although the observed correlations were of moderate magnitude (r ≈ 0.30–0.39), such effect sizes are commonly reported in psychosocial research and may still be clinically meaningful at the population level. Even modest associations between climate change worry and psychological distress can translate into a substantial public health burden when experienced by large numbers of pregnant women. These results support previous research emphasizing the psychological burden associated with climate-related worry among pregnant women [8,9,16,18,19,52,53,54,55]. For example, Kartal and Güncü (2025) reported a positive association between climate-related worry and depressive symptoms in pregnant women, while Lykins et al. (2024) identified a relationship between climate change anxiety and prenatal depression and anxiety [18,19]. Although most of these studies employed related constructs such as climate change anxiety, their findings similarly underscore the adverse mental health correlates of climate-related worry during pregnancy. Additionally, qualitative studies indicate that environmental stressors, including climate change and natural disasters, may elicit emotional reactions such as fear and anxiety during pregnancy, thereby negatively affecting maternal mental health [16,52,55].

Notably, women who responded “no opinion” regarding whether their residential area would be affected by climate change exhibited higher depression scores compared to other groups. This finding is particularly noteworthy, as the “no opinion” response may not simply reflect a lack of knowledge, but rather a distinct psychological profile characterized by emotional disengagement, uncertainty, or information-avoidant coping. Previous research suggests that individuals who feel overwhelmed by complex or threatening information may cope by disengaging or avoiding the topic altogether, a pattern that has been associated with higher levels of depressive symptoms. In the context of pregnancy—a period marked by heightened emotional sensitivity and future-oriented concerns—such avoidance or disengagement from climate-related threats may signal increased psychological vulnerability. Therefore, this group may represent a high-risk subgroup whose elevated depression levels reflect not only climate-related worry but also maladaptive coping processes in response to perceived uncontrollable stressors [56,57,58]. However, given the cross-sectional nature of the study, these interpretations remain speculative and should be examined in future longitudinal research.

Overall, these findings indicate that pregnant women experiencing higher levels of climate change worry may represent a particularly vulnerable subgroup with respect to psychological distress. Future research should employ longitudinal designs, control for pre-existing mental health conditions, and consider contextual factors such as direct environmental exposure, media consumption, and social support to better elucidate the mechanisms underlying these associations.

## 5. Conclusions

In conclusion, the findings of this study demonstrate that climate change worry, as measured by the CCWS, is significantly associated with symptoms of depression, anxiety, and stress among pregnant women. Both the present findings and previous research suggest that increased awareness of climate change-related worry and its potential mental health implications should be considered within clinical practice [3]. In this context, healthcare providers—particularly midwives and nurses—should incorporate environmental stressors such as climate change into routine psychosocial assessment processes and adapt counseling and support services for pregnant women accordingly [9,59].

## 6. Study Limitations

This study has several important limitations that should be considered when interpreting the findings. First, the cross-sectional design precludes any conclusions regarding causality or the directionality of the observed associations. Although significant relationships were identified between climate change worry and symptoms of depression, anxiety, and stress, it is not possible to determine whether climate change worry contributes to psychological distress, whether preexisting mental health vulnerability increases climate-related worry during pregnancy, or whether both are influenced by unmeasured third variables.

Second, the use of a convenience sampling method and the recruitment of participants from a single training and research hospital in one geographic region of Türkiye limit the representativeness and generalizability of the findings. Pregnant women in this specific cultural, socioeconomic, and environmental context may differ from those in other regions of the country, particularly urban centers or areas more directly exposed to climate-related hazards. Therefore, the results should be interpreted as preliminary and context-specific.

Third, the study relied on self-report instruments, which may be subject to response bias, recall bias, and social desirability effects. Participants’ reported levels of climate change worry and psychological symptoms may not fully reflect their actual experiences. In addition, baseline or pre-pregnancy mental health status was not assessed, limiting the ability to determine whether the observed psychological symptoms were specifically related to climate change worry or reflected preexisting conditions.

Furthermore, several potentially important confounding variables were not measured or controlled for in the analysis. These include direct exposure to climate-related events (such as floods, droughts, or extreme heat), frequency and type of media exposure related to climate change, individual coping styles, and levels of perceived social support. The absence of these variables restricts the ability to isolate the unique contribution of climate change worry to psychological distress.

Finally, although the sample size was adequate to detect the observed associations, the study employed primarily bivariate analyses. Future research should incorporate longitudinal designs and multivariate analytical approaches to better account for confounding factors, clarify causal pathways, and identify potential moderators and protective factors influencing the relationship between climate change worry and mental health outcomes during pregnancy.

## Figures and Tables

**Table 1:** Distribution of participants’ descriptive characteristics (*N* = 367).

Descriptive Characteristics	*n*	%
**Age**		
18–25	111	30.2
26–35	200	54.5
36–45	56	15.3
**Educational level**		
Primary school	82	22.3
Secondary school	89	24.3
High school	103	28.1
University and above	93	25.3
**Economic income status**		
Income less than expenses	121	33.0
Income equal to expenses	208	56.7
Income more than expenses	38	10.4
**Gestational week**		
First trimester	44	12.0
Second trimester	102	27.8
Third trimester	221	60.2
**Number of pregnancies**		
First	148	40.3
2–3	155	42.2
4 or more	64	17.4
**Concern about climate change**		
None	177	48.2
Low	99	27.0
Moderate	68	18.5
High	23	6.3
**Expectation that region will be affected by climate change**		
I do not think it will be affected	138	37.6
Somewhat	149	40.6
I think it will be highly affected	38	10.4
No opinion	42	11.4

**Table 2:** Mean scores of participants on the CCWS and DASS-21 *(N* = 367).

Scale Total and Subscale	Mean ± SD	Observed Range	Possible Range
Anxiety	14.26 ± 5.686	7–35	7–35
Feeling of helplessness	5.96 ± 2.806	3–15	3–15
The total CCWS score	20.22 ± 8.202	10–50	10–50
Depression	3.84 ± 3.850	0–17	0–21
Anxiety	6.10 ± 4.188	0–20	0–21
Stress	5.12 ± 4.578	0–21	0–21

Note. Observed range = the minimum and maximum scores actually obtained by participants in this study. Possible range = the theoretical minimum and maximum scores possible on the scale.

**Table 3:** Comparison of the mean rank scores of CCWS and its subscales according to participants’ descriptive characteristics (Kruskal–Wallis-H Test).

CCWS Subscales and Total Score	Variable		*n*	Mean Rank	df	χ^2^	*p*	ϵ^2^
Anxiety	Age	18–25 ^a^	111	172.48	2	5.380	0.068	-
		26–35 ^a^	200	195.54				
		36–45 ^a^	56	165.63				
Feeling of helplessness	Age	18–25 ^a^	111	178.60	2	0.707	0.702	-
		26–35 ^a^	200	188.20				
		36–45 ^a^	56	179.71				
CCWS total	Age	18–25 ^a^	111	174.46	2	3.820	0.148	-
		26–35 ^a^	200	193.71				
		36–45 ^a^	56	168.24				
Anxiety	Educational level	Primary school ^a^	82	165.21	3	16.720	0.001	0.046
		Secondary school ^a^	89	162.97				
		High school ^a^	103	184.86				
		University or higher ^b^	93	219.75				
Feeling of helplessness	Educational level	Primary school ^a^	82	169.06	3	13.712	0.003	0.037
		Secondary school ^a^	89	167.66				
		High school ^a^	103	179.43				
		University or higher ^b^	93	217.87				
CCWS total	Educational level	Primary school ^a^	82	164.88	3	16.930	0.001	0.046
		Secondary school ^a^	89	163.93				
		High school ^a^	103	183.58				
		University or higher ^b^	93	220.53				
Anxiety	Economic income status	Income less than expenses ^a^	121	167.57	2	7.459	0.024	0.020
		Income equal to expenses ^ab^	208	186.99				
		Income more than expenses ^b^	38	219.92				
Feeling of helplessness	Economic income status	Income less than expenses ^a^	121	174.79	2	5.739	0.057	-
		Income equal to expenses ^a^	208	182.57				
		Income more than expenses ^a^	38	221.16				
CCWS total	Economic income status	Income less than expenses ^a^	121	168.63	2	7.308	0.026	0.020
		Income equal to expenses ^a^	208	186.16				
		Income more than expenses ^b^	38	221.14				
Anxiety	Gestational week	First trimester ^a^	44	164.02	2	3.504	0.173	-
		Second trimester ^a^	102	198.10				
		Third trimester ^a^	221	181.47				
Feeling of helplessness	Gestational week	First trimester ^a^	44	158.67	2	2.946	0.229	-
		Second trimester ^a^	102	188.98				
		Third trimester ^a^	221	186.75				
CCWS total	Gestational week	First trimester ^a^	44	161.48	2	3.267	0.195	-
		Second trimester ^a^	102	195.77				
		Third trimester ^a^	221	183.05				
Anxiety	Number of pregnancies	First ^a^	148	196.70	2	5.312	0.070	-
		2–3 ^a^	155	181.47				
		4 or more ^a^	64	160.74				
Feeling of helplessness	Number of pregnancies	First ^a^	148	193.29	2	2.041	0.360	-
		2–3 ^a^	155	179.13				
		4 or more ^a^	64	174.31				
CCWS total	Number of pregnancies	First ^a^	148	196.31	2	4.478	0.107	-
		2–3 ^a^	155	180.56				
		4 or more ^a^	64	163.85				
Anxiety	Concern about climate change	None ^a^	177	153.81	3	45.262	0.000	0.124
		Low ^a^	99	182.39				
		Moderate ^b^	68	237.62				
		High ^b^	23	264.72				
Feeling of helplessness	Concern about climate change	None ^a^	177	158.95	3	41.663	0.000	0.114
		Low ^a^	99	174.49				
		Moderate ^b^	68	234.21				
		High ^b^	23	269.24				
CCWS total	Concern about climate change	None ^a^	177	154.07	3	47.052	0.000	0.129
		Low ^a^	99	180.14				
		Moderate ^b^	68	239.37				
		High ^b^	23	267.24				
Anxiety	Expectation that region will be affected by climate change	I do not think it will be affected ^a^	138	141.58	3	52.063	0.000	0.142
		Somewhat ^b^	149	202.73				
		I think it will be highly affected ^c^	38	270.05				
		No opinion ^a^	42	179.07				
Feeling of helplessness	Expectation that region will be affected by climate change	I do not think it will be affected ^a^	138	143.36	3	46.220	0.000	0.126
		Somewhat ^b^	149	198.05				
		I think it will be highly affected ^c^	38	264.53				
		No opinion ^a^	42	194.83				
CCWS total	Expectation that region will be affected by climate change	I do not think it will be affected ^a^	138	140.27	3	53.035	0.000	0.145
		Somewhat ^b^	149	202.78				
		I think it will be highly affected ^c^	38	269.62				
		No opinion ^a^	42	183.61				

Footnote: Groups sharing the same superscript letter do not differ significantly. 0.01 ≤ η^2^ < 0.06: “small”, 0.06 ≤ η^2^ < 0.14: “medium”, η^2^ ≥ 0.14: “large” [33].

**Table 4:** Comparison of the mean rank scores of DASS-21 according to participants’ descriptive. (Kruskal–Wallis-H Test).

DASS-21 Subscale	Variable		*n*	Mean Rank	Df	χ^2^	*p*	*ϵ* ^2^
Depression	Age	18–25 ^a^	111	198.13	2	4.676	0.097	-
		26–35 ^a^	200	173.23				
		36–45 ^a^	56	194.48				
Anxiety	Age	18–25 ^a^	111	187.88	2	1.498	0.473	-
		26–35 ^a^	200	178.36				
		36–45 ^a^	56	196.47				
Stress	Age	18–25 ^a^	111	198.54	2	3.050	0.218	-
		26–35 ^a^	200	177.22				
		36–45 ^a^	56	179.39				
Depression	Educational level	Primary school ^ab^	82	178.68	2	14.132	0.003	0.039
		Secondary school ^a^	89	154.48				
		High school ^b^	103	210.98				
		University or higher ^ab^	93	187.06				
Anxiety	Educational level	Primary school ^a^	82	173.79	2	1.232	0.745	-
		Secondary school ^a^	89	185.49				
		High school ^a^	103	190.99				
		University or higher ^a^	93	183.83				
Stress	Educational level	Primary school ^a^	82	179.63	2	3.902	0.272	-
		Secondary school ^a^	89	168.43				
		High school ^a^	103	197.53				
		University or higher ^a^	93	187.76				
Depression	Economic income status	Income less than expenses ^a^	121	177.60	2	0.729	.694	-
		Income equal to expenses ^a^	208	186.47				
		Income more than expenses ^a^	38	190.89				
Anxiety	Economic income status	Income less than expenses ^a^	121	167.00	2	5.111	0.078	-
		Income equal to expenses ^a^	208	194.29				
		Income more than expenses ^a^	38	181.78				
Stress	Economic income status	Income less than expenses ^a^	121	179.57	2	0.950	0.622	-
		Income equal to expenses ^a^	208	188.46				
		Income more than expenses ^a^	38	173.70				
Depression	Gestational week	First trimester ^a^	44	179.09	2	4.084	0.130	-
		Second trimester ^a^	102	201.81				
		Third trimester ^a^	221	176.76				
Anxiety	Gestational week	First trimester ^a^	44	188.43	2	2.233	0.327	-
		Second trimester ^a^	102	196.08				
		Third trimester ^a^	221	177.54				
Stress	Gestational week	First trimester ^a^	44	187.81	2	0.198	0.906	-
		Second trimester ^a^	102	186.62				
		Third trimester ^a^	221	182.03				
Depression	Number of pregnancies	First ^a^	148	184.51	2	0.120	0.942	-
		2–3 ^a^	155	182.12				
		4 or more ^a^	64	187.38				
Anxiety	Number of pregnancies	First ^a^	148	201.03	2	7.358	0.025	0.020
		2–3 ^b^	155	176.91				
		4 or more ^b^	64	161.79				
Stress	Number of pregnancies	First^a^	148	201.95	2	7.336	0.026	0.020
		2–3 ^b^	155	170.17				
		4 or more^ab^	64	176.00				
Depression	Concern about climate change	None ^a^	177	179.55	3	2.303	0.512	-
		Low ^a^	99	184.38				
		Moderate ^a^	68	184.61				
		High ^a^	23	214.80				
Anxiety	Concern about climate change	None ^a^	177	174.09	3	3.535	0.316	-
		Low ^a^	99	189.23				
		Moderate ^a^	68	194.46				
		High ^a^	23	206.85				
Stress	Concern about climate change	None ^a^	177	178.56	3	2.492	0.477	-
		Low ^a^	99	180.03				
		Moderate ^a^	68	200.46				
		High ^a^	23	194.30				
Depression	Expectation that region will be affected by climate change	I do not think it will be affected ^a^	138	171.32	3	13.911	0.003	0.038
		Somewhat ^a^	149	181.04				
		I think it will be highly affected ^a^	38	180.36				
		No opinion ^b^	42	239.48				
Anxiety	Expectation that region will be affected by climate change	I do not think it will be affected ^a^	138	170.18	3	7.690	0.053	-
		Somewhat ^a^	149	182.85				
		I think it will be highly affected ^a^	38	218.64				
		No opinion ^a^	42	202.17				
Stress	Expectation that region will be affected by climate change	I do not think it will be affected ^a^	138	170.26	3	8.356	0.039	0.023
		Somewhat ^a^	149	185.43				
		I think it will be highly affected ^ab^	38	184.37				
		No opinion ^b^	42	223.75				

Footnote: Groups sharing the same superscript letter do not differ significantly. 0.01 ≤ η^2^ < 0.06: “small”, 0.06 ≤ η^2^ < 0.14: “medium”, η^2^ ≥ 0.14: “large” [33].

**Table 5:** Correlation between participants’ CCWS and DASS-21 scores (Spearman’s rho).

CCWS Subscales and Total Score		Depression (DASS-21)	Anxiety (DASS-21)	Stress (DASS-21)
Anxiety	r	0.326 **	0.390 **	0.318 **
	*p*	0.000	0.000	0.000
Feeling of helplessness	r	0.373 **	0.334 **	0.298 **
	*p*	0.000	0.000	0.000
The total CCWS score	r	0.360	0.387	0.327
	*p*	0.000	0.000	0.000

** Correlation is significant at the 0.01 level (two-tailed).

## Data Availability

The raw data supporting the conclusions of this article will be made available by the authors on request.
